# Discovery of Potential GPRC5D Inhibitors through Virtual Screening and Molecular Dynamics Simulations

**DOI:** 10.1002/open.202500360

**Published:** 2025-09-07

**Authors:** Xi Chen, Xinle Yang, Roufen Chen, Lei Xu, Xiaowu Dong, Zhen Cai

**Affiliations:** ^1^ Bone Marrow Transplantation Center the First Affiliated Hospital Zhejiang University School of Medicine Hangzhou Zhejiang 310003 China; ^2^ Lymphoma Department Zhejiang Cancer Hospital Hangzhou Zhejiang 310022 China; ^3^ College of Pharmaceutical Sciences Zhejiang University of Technology Hangzhou 310014 China; ^4^ College of Pharmaceutical Sciences Zhejiang University Hangzhou 310058 China; ^5^ Institute of Bioinformatics and Medical Engineering School of Electrical and Information Engineering Jiangsu University of Technology Changzhou 213001 P. R. China; ^6^ Institute of Hematology Zhejiang University Hangzhou Zhejiang 310058 China

**Keywords:** deep learning, GPRC5D, molecular dynamics, virtual screening

## Abstract

G protein‐coupled receptor family C, group 5, member D (GPRC5D), a member of the G protein‐coupled receptor (GPCR) family, has recently emerged as a promising target for immunotherapy in hematologic malignancies, particularly multiple myeloma. However, no systematic virtual screening studies have been conducted to identify small‐molecule inhibitors targeting GPRC5D. To address this gap, a multistep computational screening strategy is developed that integrates Protein−Ligand Affinity prediction NETwork (PLANET), a GPU‐accelerated version of AutoDock Vina (Vina‐GPU), molecular mechanics/generalized born surface area (MM/GBSA), and an online tool for Absorption, Distribution, Metabolism, Excretion, and Toxicity (ADMET) property prediction (admetSAR 3.0), complemented by molecular dynamics (MD) simulations and absolute binding free energy (ABFE). From an initial library of 8,617 compounds, four candidates (compounds 1, 2, 7, and 8) are prioritized. Among them, compound 2 shows relatively strong binding affinity (MM/GBSA Δ*G* = −79.8 kcal mol^−1^, ABFE = −9.0 kcal mol^−1^) and high drug‐likeness (quantitative estimate of drug‐likeness = 0.670). MD simulations confirm its stable salt bridge interactions with key residues ASP238 and ASP239. This study proposes a systematic virtual screening workflow to facilitate the discovery of GPRC5D‐targeted therapeutics.

## Graphical abstract

A systematic computational workflow integrates Protein–Ligand Affinity prediction NETwork (PLANET), a GPU‐accelerated version of AutoDock Vina (Vina‐GPU), molecular mechanics/generalized born surface area (MM/GBSA), absorption distribution metabolism excretion toxicity prediction (admetSAR 3.0), molecular dynamics (MD), and absolute binding free energy (ABFE) calculations to identify potential G protein‐coupled receptor family C group 5 member D (GPRC5D) inhibitors for multiple myeloma therapy.

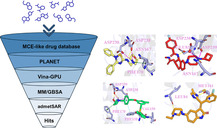

## 1 Introduction

Multiple myeloma is a genetically complex blood cancer that accounts for ≈10% of hematologic malignancies and has a 5‐year survival rate of around 60%.^[^ ^1^ ^,^ ^2^ ^]^ Multiple myeloma affects multiple organs, causing fatigue, pain, mobility issues, neurological symptoms, kidney problems, bone disease, and frequent infections.^[^ 3, 4, 5 ^]^ Despite advances in B‐cell maturation antigen (BCMA)‐targeted therapies, bispecific antibodies, and CAR‐T therapies, patients still face cycles of remission and relapse, with each new line of therapy often becoming less effective due to toxicity and comorbidities.^[^ 6, 7, 8 ^]^ Currently, the G protein‐coupled receptor class C group 5 member D (GPRC5D) has been reported as a promising avenue in the treatment of Multiple myeloma.^[^ ^9^ ^]^

**Figure 1:**
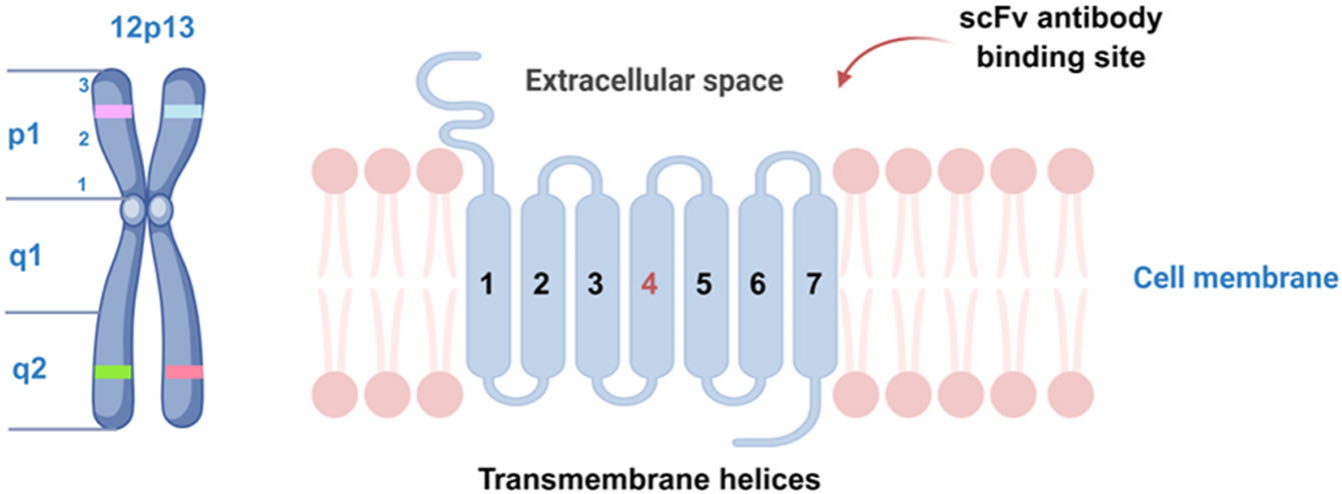
Chromosomal localization and predicted protein structure of GPRC5D.

GPRC5D, located on human chromosome 12p13 (**Figure** **1**), is a G protein‐coupled receptor classified as an orphan due to its unidentified endogenous ligand.^[^ ^10^ ^]^ It belongs to mammalian GPCR class C group 5 (GPRC5) receptor family, which includes four members: GPRC5A (RAIG1), GPRC5B (RAIG2), GPRC5C (RAIG3), and GPRC5D.^[^ ^11^ ^]^ Expression of GPRC5D protein has been observed in immune cells and the epithelial tissues of the skin and tongue.^[^ ^12^ ^]^ In immune cells, GPRC5D is predominantly expressed in plasma cells, while its expression is minimal or absent in normal B cells, T cells, natural killer cells, monocytes, granulocytes, and bone marrow progenitor cells.^[^ 13, 14, 15 ^]^ Based on a previous study, GPRC5D mRNA levels are significantly elevated in Multiple myeloma cells compared to other hematologic malignancies. The selective expression in Multiple myeloma cells suggests that GPRC5D is an ideal target for immune effector cell‐mediated therapy to treat Multiple myeloma.^[^ ^15^ ^,^ ^16^ ^]^

Therapies targeting GPRC5D focus on monoclonal antibodies and CAR‐T cells, effectively targeting and eliminating myeloma cells expressing this receptor.^[^ ^17^ ^,^ ^18^ ^]^ However, despite the significant efficacy demonstrated by immunotherapy strategies such as CAR‐T therapy, their high cost, complex production processes, and potential toxicities, such as cytokine release syndrome, limit their widespread accessibility. Therefore, exploring GPRC5D as a target for alternative treatment modalities beyond CAR‐T therapy, particularly the development of small‐molecule inhibitors, holds significant research and clinical value. Compared to cell therapy, small‐molecule drugs offer advantages such as oral administration, lower costs, well‐defined synthesis pathways, and controllable pharmacokinetics, making them suitable for a wide range of patients and easier to combine with other medications.

Recently, the structure of GPRC5D has been resolved, providing a foundation for the discovery of potential molecular inhibitors targeting GPRC5D. The structural findings reveal that GPRC5D closely resembles typical class C GPCRs in the transmembrane region. A unique head‐to‐head homodimer arrangement was identified, mainly involving TM4, which sets GPRC5D apart from other class C homo‐ or heterodimers. Additionally, the binding site for a preclinical single‐chain antibody (scFv), involving a substantial extracellular domain of GPRC5D, has been characterized (Figure 1). However, the specific type of downstream G‐protein binding could not be identified, and the precise activation mechanism remains elusive. Key motifs associated with activation, typically found in Class C GPCRs, are absent, and GPRC5D is still classified as an orphan receptor without identified endogenous ligands, possibly due to its inactive state.^[^ ^19^ ^]^

Although these structural insights are valuable, no cocrystallized small molecules or well‐defined ligand‐binding pockets have been reported to date. Furthermore, there are no known bioactive small‐molecule modulators targeting GPRC5D. These limitations pose significant challenges for structure‐based virtual screening, particularly for ligand‐based strategies that rely on known active compounds for validation, benchmarking, or redocking. To address these challenges, we integrated the complementary strengths of computer‐aided drug design (CADD) and artificial intelligence‐driven drug design (AIDD) to develop a multistep virtual screening strategy tailored to the unique structural and pharmacological characteristics of GPRC5D. Through virtual screening and mechanism‐based simulations, our approach not only enables the identification of potential lead compounds but also provides critical tools and theoretical groundwork for functional studies and targeted drug discovery. To the best of our knowledge, this work represents the first systematic and comprehensive attempt to explore GPRC5D as a small‐molecule drug target using an integrated CADD‐AIDD framework.

## 2 Experimental Section

### 2.1 Protein Structure Preparation

The human GPRC5D protein sequence (UniProt ID: Q9NZD1) was retrieved from the UniProt database to confirm the full‐length sequence and assess domain completeness in available structural entries.^[^ ^20^ ^]^ According to the UniProt structure annotations, two resolved structures were available: PDB ID 8YZK (3.40 Å)^[^ ^19^ ^,^ ^21^ ^]^ and PDB ID 9IMA (2.65 Å).^[^ ^22^ ^,^ ^23^ ^]^

Based on its higher resolution and more complete coverage, we selected PDB 9IMA as the structural model for this study. The corresponding cryo‐EM structure was downloaded from the RCSB Protein Data Bank (www.rcsb.org).^[^ ^24^ ^]^ The protein structure was subsequently processed using the Protein Preparation Wizard in Schrödinger 2021–2,^[^ ^25^ ^]^ including bond order assignment, hydrogen addition, optimization of protonation states (via Epik at pH 7.0 ± 2.0),^[^ ^26^ ^]^ and energy minimization with the OPLS4 force field.^[^ ^27^ ^]^ To better capture the conformational flexibility and realistic binding pocket conformation, molecular dynamics (MD) simulations were later performed (see Section 2.8 for details).

### 2.2 Ligand Database Preparation

The compound database used for virtual screening was derived from the MedChemExpress (MCE) drug‐like database. To ensure the suitability of compounds for molecular docking and screening, preprocessing was conducted using the LigPrep^[^ ^28^ ^]^ module in Schrödinger 2021–2. During preprocessing, default parameters were applied, including energy optimization using the OPLS4 force field and protonation state generation within a target pH range of 7.0 ± 2.0. Additionally, the Epik module was utilized for salt removal and generation of relevant tautomeric and ionization states, ensuring accurate chemical representations within a physiological pH range (7.0 ± 2.0). Importantly, duplicate compounds were identified and removed to ensure structural uniqueness and avoid redundancy in the virtual screening process.

### 2.3 Binding Pocket Prediction

In this study, we employed the SiteMap^[^ ^29^ ^]^ module in Schrödinger software to predict the binding pocket of the target protein GPRC5D. SiteMap identifies potential small molecule binding sites by analyzing the geometric and physicochemical properties of the protein surface, assigning a score (SiteScore) to each predicted site. To ensure a biologically relevant conformation, the final frame of the protein structure was extracted from the MD simulation trajectory and used as the input for binding pocket identification, with default parameter settings applied. The predicted binding pockets (coordinates: [X: −0.19, Y: 4.49, Z: −19.54]) were then visualized in three dimensions within the Maestro interface to facilitate further analysis.

### 2.4 Receptor Grid Generation

The receptor grid generation was performed using the Glide module within the Schrödinger software to define the spatial extent of the protein binding pocket.^[^ ^30^ ^]^ First, the final frame of the protein structure from the MD simulation trajectory was imported into the Maestro interface and preprocessed. Subsequently, using the Receptor Grid Generation tool in the Glide module, the spatial range of the binding pocket was defined, centered on the coordinates of the binding pocket predicted by SiteMap. The specific parameters for grid generation were set as follows: the grid box size was set to 20 × 20 × 20 Å^3^ to ensure complete coverage of the binding pocket and its surrounding regions; the van der Waals scaling factor and partial charge cutoff were set to their default values of 1.0 and 0.25, respectively. The generated grid files were used for subsequent virtual screening to accurately evaluate the affinity between ligands and the receptor.

### 2.5 Virtual Screening Workflow

Virtual screening was conducted using Protein‐Ligand Affinity Prediction NETwork^[^ ^31^ ^]^ and GPU‐accelerated version of AutoDock Vina (Vina‐GPU).^[^ ^32^ ^,^ ^33^ ^]^ Molecular docking was initially performed using the PLANET, which utilizes a graphical representation of the 3D structure of the GPRC5D binding pocket and the SMILES representation of ligand molecules as inputs. The planet.py script was executed to perform the affinity predictions. The PLANET model was obtained from GitHub (available at: https://github.com/ComputArtCMCG/PLANET/).

Following this, we utilized Vina‐GPU—a GPU‐accelerated molecular docking tool developed based on AutoDock Vina—to perform molecular docking on the 1,694 compounds selected from the initial screening. The Vina‐GPU parameters were configured as follows: a docking search space cubic dimension of 20 × 20 × 20 Å^3^, the exhaustiveness parameter was set to 32, and the thread count was set to 8000, to further optimize the selection of potential GPRC5D inhibitors.

### 2.6 MM/GBSA Binding Free Energy Calculation

Molecular mechanics/generalized born surface area (MM/GBSA) is a computational method. It combines molecular mechanics (MM) and generalized born (GB) solvent models for estimating the free energy changes during protein−ligand binding.^[^ ^34^ ^]^ Initially, the molecules obtained by Vina‐GPU screening were prepared using the LigPrep module in Schrödinger. Then, the receptor grid file of GPRC5D and the prepared ligand file were loaded into the system using the Ligand Docking module, and the docking was run under the default settings by selecting the OPLS4 force field and SP precision.^[^ ^35^ ^]^ After docking was completed, we used Schrödinger's Prime MM/GBSA module to perform free energy calculations of protein–ligand binding, where we set the solvent model to VSGB,^[^ ^36^ ^]^ the force field to OPLS4, and modified the flexible residue distances in protein flexibility to 4.0 Å from the ligand, to perform the binding free energy calculations for the lowest energy state reached by the molecular structure. The binding free energy was calculated as

(1) $$\Delta G_{\mathrm{bind}}=\Delta E_{\mathrm{MM}}+\Delta G_{\mathrm{solv}}+\Delta G_{\mathrm{SA}}$$

where Δ*G*_bind_ represents the free energy change upon binding of protein and ligand, and is used to assess the binding affinity between the molecules. Δ*E*_MM_ represents the molecular mechanics energy difference between the ligand in solution and complexed in the protein, Δ*G*_solv_ represents the energy contribution of the solvent molecules to the solvation of the protein−ligand complex, and Δ*G*_SA_ is the change in energy resulting from the surface area.

### 2.7 ADMET Property Prediction via admetSAR 3.0

The admetSAR 3.0^[^ ^37^ ^]^ is an integrated Absorption, Distribution, Metabolism, Excretion, and Toxicity (ADMET) prediction platform developed based on machine learning algorithms. It can quickly output multidimensional parameterized evaluation results by inputting the SMILES string of a compound. Specifically, the lipophilicity (LogP) and ionization constant (pKa) are selected to characterize the fundamental properties of the compound, while the blood‐brain barrier permeability and the quantitative estimate of drug‐likeness (QED) are used to predict drug delivery and metabolic potential. Additionally, the median toxic dose (T50) and drug‐induced liver injury risk (DILI) are employed to assess safety thresholds, thereby providing comprehensive support for the evaluation of compound druggability and risk prediction.

### 2.8 MD Simulation Protocol

In this study, we employed MD simulations to obtain the stable conformations of the GPRC5D protein and predict the binding modes of compounds with it. Specifically, we utilized the System Builder and Molecular Dynamics modules within the Schrödinger 2021–2 software suite for the simulations. As GPRC5D is a membrane protein, we first constructed the membrane system using the System Builder module. The POPC membrane model (300 K) was adopted, and the membrane environment was established through automatic placement and alignment procedures. Subsequently, we selected the TIP3P^[^ ^38^ ^]^ water model as the solvent system and employed an orthorhombic unit cell as the boundary condition for the simulation system, with a 10.0 Å buffer region set outside the molecular boundary. To mimic physiological conditions, 0.15 M NaCl was added to the system, and additional Cl^−^ counterions were introduced to neutralize the net system charge.

For the simulation of the apo GPRC5D protein (without ligand), 3 Cl^−^ ions were added to achieve electrostatic neutrality, resulting in a system containing 31 674 atoms.6 Cl^−^ ions for the protein−compound **1** system; 5 Cl^−^ ions each for the protein−compound **2**, **4**, **5**, **7**, **8**, and **9** systems; 7 Cl^−^ ions for the protein−compound **3** system; and 4 Cl^−^ ions each for the protein−compound **6** and **10** systems. The final constructed systems consisted of the following number of atoms: protein‐compound **1** (31 838 atoms), protein‐compound **2** (31 902 atoms), protein−compound **3** (31 847 atoms), protein−compound **4** (31 906 atoms), protein−compound **5** (31 572 atoms), protein−compound **6** (31 841 atoms), protein−compound **7** (31 828 atoms), protein‐compound **8** (31 812 atoms), protein–compound **9** (31 939 atoms), and protein‐compound **10** (31 800 atoms). After system construction, we first performed energy minimization to remove steric clashes and unfavorable contacts. This was followed by a two‐stage equilibration process: (a) Constant Number of particles, Volume, and Temperature  (NVT) equilibration for 1 ns, under constant volume and temperature (300 K), allowing the system to reach thermal equilibrium. (b) Constant Number of particles, Pressure, and Temperature (NPT) equilibration for 1 ns, under constant pressure (1.01325 bar) and temperature (300 K), to stabilize the system's density and pressure. The temperature of 300 K was chosen as it is a widely accepted standard in MD simulations to approximate physiological conditions, while also providing improved numerical stability and compatibility with the OPLS4 force field. Following equilibration, 500 ns production MD simulations were performed for each protein–ligand complex under the NPT ensemble using the OPLS4 force field. Simulation snapshots were saved every 500 ps, and energy data were recorded every 1.2 ps. Hydrogen bond occupancy rates were subsequently analyzed using the VMD software to assess the interaction persistence between the compounds and the GPRC5D protein.

The above methodological research proposal and its detailed description, as well as the software tools used throughout the study, are presented in **Table** **1**.

**Table 1:** The research proposals, detailed descriptions, and software tools utilized throughout the investigation process.

Proposal	Description	Workshops
Protein structure preparation	Structure optimization	Schrödinger
	Relax conformation	Schrödinger
Virtual screening	Affinity prediction	PLANET
	Molecular docking	Vina‐GPU
	MM/GBSA	Schrödinger
	Screening compounds with favorable ADMET properties	admetSAR 3.0
MD simulation	Analysis of binding modes of complexes	Schrödinger
ABFE calculation	Estimation of absolute binding free energy	AI2Physic platform

### 2.9 Absolute Binding Free Energy (ABFE) Prediction

In this study, the AI2Physic platform was used to predict the ABFE of candidate compounds. The platform is based on a self‐developed deep learning model, LumiNet, which integrates physical knowledge, distance distribution features, and geometric information to model protein—ligand complexes and evaluate their binding free energies. All protein–ligand systems were automatically preprocessed according to the platform's standard workflow. During the prediction process, the LumiNet model directly outputs the ABFE of each complex by modeling atomic pair distance distributions and energy contributions.

## 3 Results and Discussion

### 3.1 Preparation of Ligand Database and GPRC5D Protein Structure

In virtual screening, based on the MCE drug‐like drug database, the preparation quality of the ligand database is a critical factor determining the reliability and efficiency of screening results. By removing duplicate molecules and standardizing molecular structures, not only can screening efficiency be enhanced, but also docking anomalies caused by structural input errors or nonstandard formats can be avoided. During the structural optimization phase, the use of the Schrödinger LigPrep module for conformational optimization and force field parameter calibration of ligand molecules effectively improves the rationality of docking conformations. Additionally, precise regulation of ligand protonation states plays a pivotal role in accurately simulating ligand‐target binding patterns.

In the protein structure preprocessing stage, systematic optimization through Schrödinger's Protein Preparation Wizard module restores the structural integrity of crystal structures by completing missing residues, optimizing hydrogen bond networks to rectify aberrant side‐chain flips, and predicting physiologically relevant protonation states of residues. Coupled with energy minimization using the OPLS4 force field to eliminate atomic clashes, this refined preprocessing constructs an energetically balanced protein model. Such meticulous preparation not only establishes a structural foundation for accurate binding pocket prediction via SiteMap but also significantly enhances the reliability of subsequent molecular docking in simulating ligand‐target binding modes and affinity predictions.

### 3.2 Binding Pocket Prediction

Since no cocrystallized ligand or annotated binding site is available for GPRC5D, binding pocket prediction was necessary to guide docking. Three potential ligand‐binding pockets were identified using SiteMap, with the highest‐scoring pocket located near residue ASP239 in the transmembrane region (see Figure S1, Supporting Information**)**. This pocket achieved a DScore of 1.046, indicating typical physicochemical properties of a druggable site and strong potential for ligand binding.

### 3.3 Virtual Screening Workflow and Hit Selection

**Figure 2:**
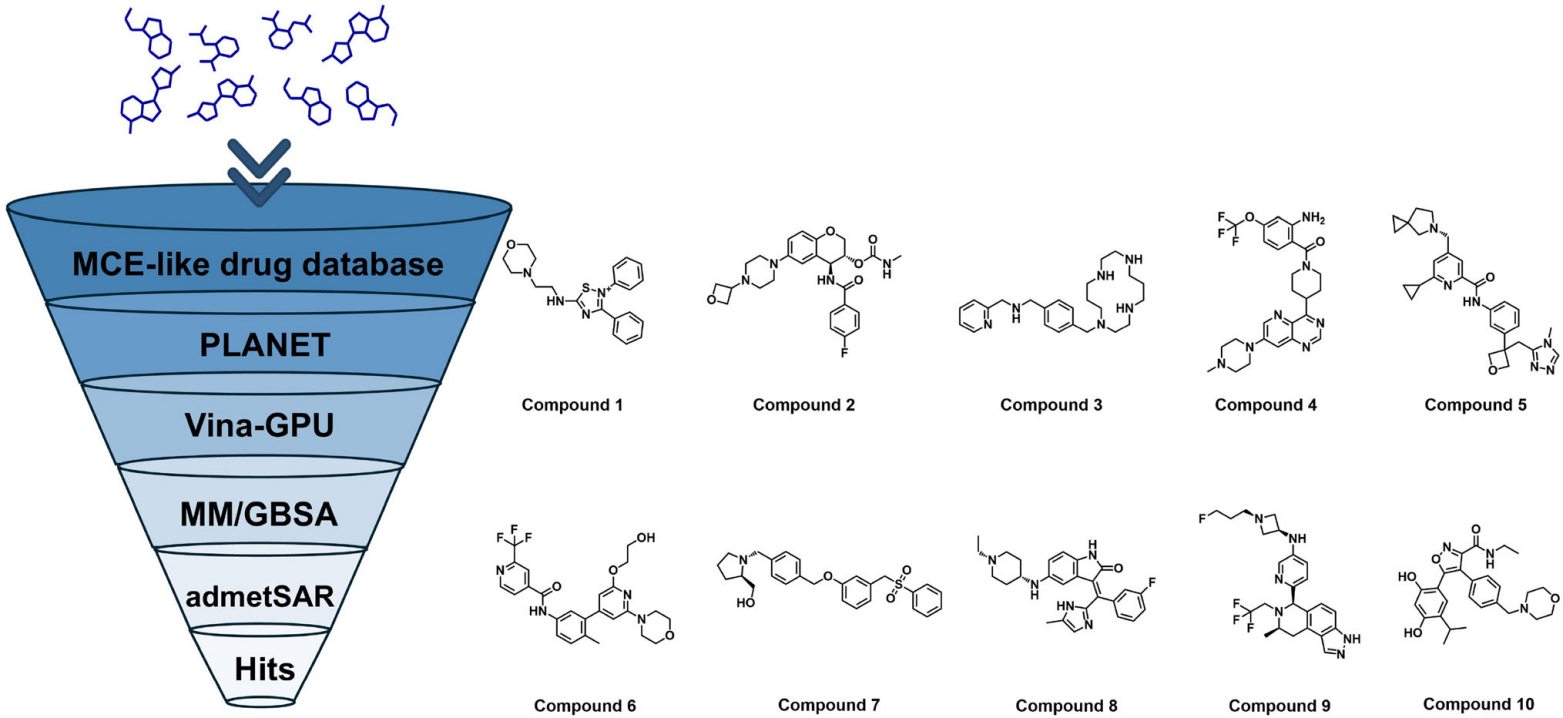
Schematic overview of the virtual screening workflow used to identify GPRC5D‐targeted small‐molecule inhibitors, along with the chemical structures of the top‐ranked candidate compounds.

Due to the absence of reported small‐molecule inhibitors targeting GPRC5D and the lack of cocrystal structures with resolved ligands or annotated binding sites, benchmarking the docking protocol or including a known active compound as a positive control was not feasible in this study. To improve the reliability and efficiency of hit identification under these constraints, we adopted a multilevel virtual screening strategy, integrating computational tools such as PLANET, Vina‐GPU, MM/GBSA, and admetSAR 3.0 (**Figure** **2**). In the virtual screening workflow, we first employed an advanced graph neural network model—PLANET. Renowned for its exceptional computational performance and predictive accuracy, PLANET can complete large‐scale virtual screening tasks in a remarkably short time while maintaining accuracy comparable to traditional molecular docking methods. Leveraging its efficiency and reliability, we used PLANET to conduct an initial screening of the compound database, from which we selected the top 1,694 compounds as candidates for the next round of screening.

In the second round of virtual screening, we utilized the Vina‐GPU software to further evaluate the binding affinity of these compounds to the GPRC5D binding pocket. Vina‐GPU scores the compounds based on their binding free energy, with lower scores indicating stronger binding affinity to the target protein. To further validate the reliability of the docking results, we employed the MM/GBSA method to calculate the binding free energy of the candidate compounds selected by Vina‐GPU. MM/GBSA, through molecular mechanics and the GBSA model, provides a more accurate estimation of binding free energy, significantly enhancing the reliability of the screening results and further supporting the potential of these compounds as candidate molecules. Based on the binding free energy ranking, we selected the top 120 molecules for subsequent analysis.

Subsequently, ADMET property prediction for these 120 molecules was conducted using the admetSAR 3.0 computational platform. Building upon this, the QED was integrated to quantitatively evaluate the drug‐like characteristics of the molecules, establishing a priority ranking criterion based on QED scores. Through multiparameter comprehensive evaluation, the top 10 molecules with the highest QED values were ultimately selected as core lead compounds (Figure 2). The scoring results of the ten selected compounds at each stage of the screening process are summarized in **Table** **2**. This screening workflow not only significantly enhanced the efficiency of drug discovery but also laid a solid foundation for subsequent experimental validation and lead compound optimization.

**Table 2:** Summary of virtual screening scores and drug‐likeness (QED) values for the top 10 candidate compounds.

Entry	PLANET Score	Vina‐GPUScore	MM/GBSA Δ*G* bind[kcal mol^−1^]	admetSAR 3.0QED Score
**Compound 1**	7.1	−7.8	−77.0	0.680
**Compound 2**	6.7	−8.1	−79.8	0.670
**Compound 3**	7.1	−7.9	−74.6	0.582
**Compound 4**	7.0	−8.5	−79.8	0.529
**Compound 5**	8.0	−8.6	−80.1	0.508
**Compound 6**	7.2	−8.8	−74.1	0.505
**Compound 7**	6.9	−8.2	−74.1	0.504
**Compound 8**	8.4	−7.8	−77.8	0.499
**Compound 9**	6.8	−7.8	−74.9	0.498
**Compound 10**	8.0	−8.7	−76.0	0.482

**Figure 3:**
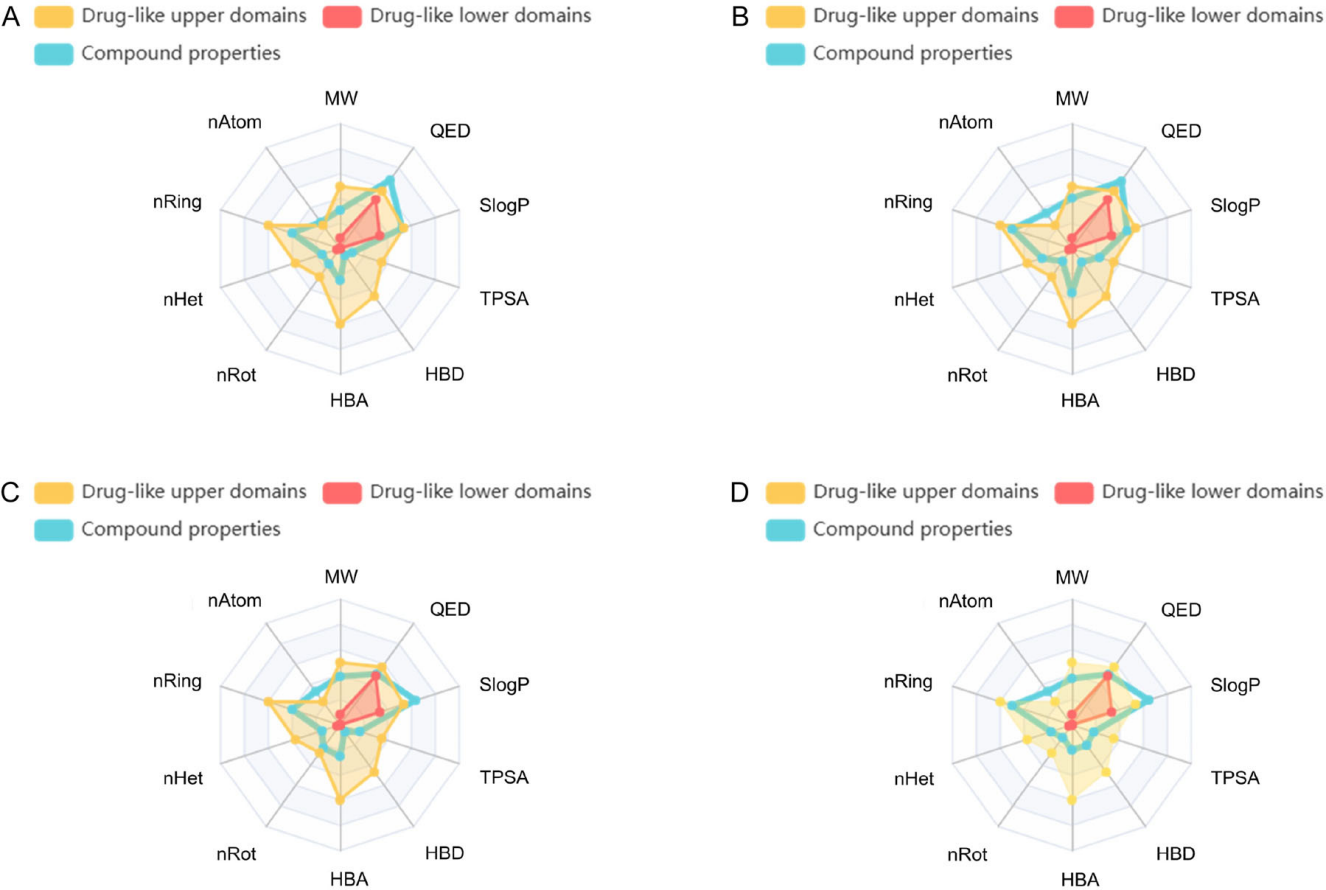
Radar charts illustrating the physicochemical property profiles of compounds **1**, **2**, **7**, and **8**. A) Compound **1**. B) Compound **2**. C) Compound **7**. D) Compound **8**.

The analysis of the chemical property radar chart for the screened compounds revealed that 70% of the compounds exhibited QED values exceeding the drug‐likeness threshold of 0.5 (**Figure** **3**, Figure S2–S7, Supporting Information). Notably, compound **1** (0.680) and compound **2** (0.670) demonstrated significantly higher QED values than other candidates, indicating superior drug‐like characteristics. Further evaluation identified that 80% of the compounds maintained molecular weights below 500 Da, a favorable feature for effective absorption through the gastrointestinal passive diffusion pathway. Additionally, all candidate molecules displayed topological polar surface area values under 140 Å^2^, suggesting an optimal balance between lipophilicity and aqueous solubility. This equilibrium facilitates transmembrane transport through moderate lipophilicity while ensuring effective dispersion in biological fluids via appropriate water solubility.

### 3.4 MD Analysis of Protein−Ligand Complexes

To further evaluate the binding stability and interaction mechanisms between the candidate compounds and the GPRC5D target, 500 ns MD simulations were performed for all 10 screened compounds. Among them, compounds **1**, **2**, **7**, and **8** exhibited notably superior binding characteristics.

**Figure 4:**
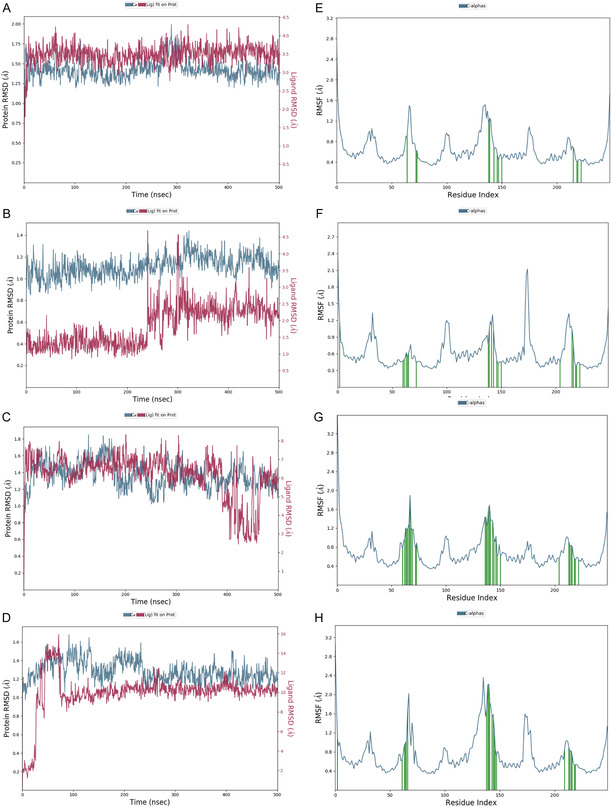
RMSD and RMSF analyses of GPRC5D‐ligand complexes with compounds **1**, **2**, **7**, and **8** over a 500 ns MD simulation. A–D) RMSD trajectories of the protein backbone (Cα atoms, blue) and ligands (red) for (A) compound **1**, (B) compound **2**, (C) compound **7**, and (D) compound 8. E–H) RMSF profiles of protein residues for (E) compound **1**, (F) compound 2, (G) compound **7**, and (H) compound **8**.

As shown in **Figure** **4**, all four compounds exhibited stable RMSD trajectories of the protein backbone (Cα atoms) throughout the simulation, with overall fluctuations maintained within the range of 1.0–2.0 Å, indicating structural stability of the complexes (Figures 4A–D). RMSF analysis further confirmed the structural stability of the complexes (Figures 4E–H). In all systems, most residues displayed low flexibility, particularly within the binding pocket. Key residues involved in ligand interactions, such as ASP238, ASP239, and ASN167, showed minimal fluctuations, suggesting that ligand binding contributed positively to the structural stabilization of the binding site.

To further investigate the dynamic effects of ligand binding, we conducted a 500 ns MD simulation of the apo form of GPRC5D under identical simulation conditions. The RMSD of the apo structure remained relatively stable throughout the trajectory, indicating overall structural stability in the absence of a ligand (Figure S8, Supporting Information). To assess local flexibility, we compared the RMSF profiles of the apo protein with those of the ligand‐bound complexes (compounds **1**, **2**, **7**, and **8**). The apo receptor exhibited notably greater fluctuations in specific regions, especially around residues 150–170 and 230–250 (Figure 4E–H and S9, Supporting Information). These differences were particularly pronounced near ASP238 and ASP239, which lie within the predicted binding pocket. Upon ligand binding, residues in the 230–250 segment showed a general decrease in fluctuation. While RMSF values in this region frequently exceeded 1.2 Å in the apo form, they were largely reduced below this threshold in most ligand‐bound complexes. This stabilization was associated with persistent polar interactions, including hydrogen bonds and salt bridges, involving ASP238, ASP239, and neighboring residues.

Building upon the confirmation that compounds **1**, **2**, **7**, and **8** exhibit favorable structural stability during the simulation, their binding conformations with GPRC5D were further analyzed in detail. To gain a more comprehensive understanding of the recognition patterns and interaction mechanisms of these candidate ligands within the binding pocket, this study employed both 2D interaction diagrams and 3D structural visualizations to intuitively illustrate the key interacting residues and their spatial arrangements in the four complexes.

**Figure 5:**
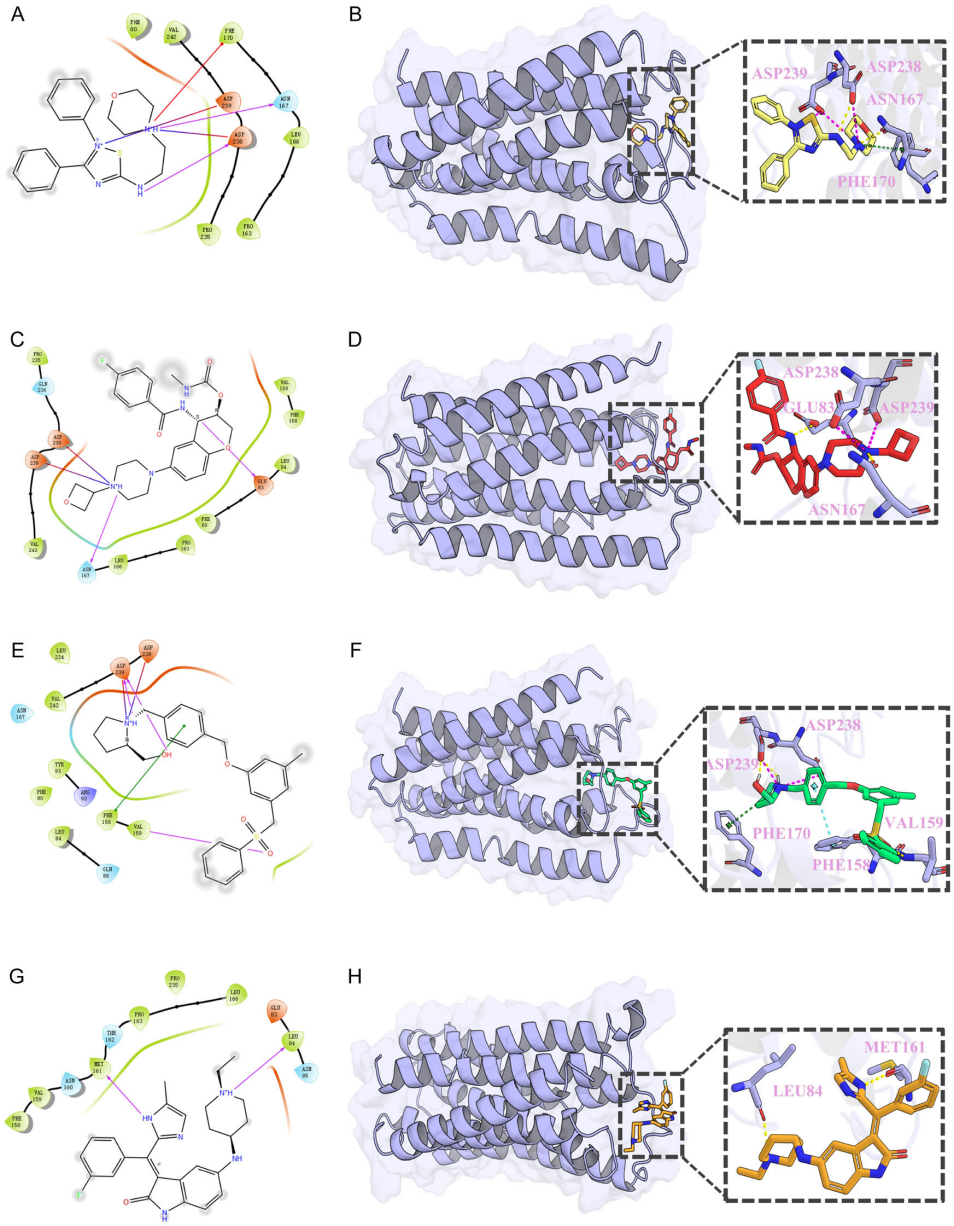
2D and 3D binding interaction diagrams of A,B) compound **1**, C,D) compound **2**, E,F) compound **7**, and G,H) compound **8** with GPRC5D. Panels A, C, E, and G show the 2D interaction maps, while panels B, D, F, and H display the corresponding 3D binding poses.

As shown in **Figure** **5**, compounds **1**, **2**, and **7** exhibited highly consistent interaction patterns with GPRC5D, with key contact residues primarily clustered around a set of conserved polar residues within the binding pocket. Among them, ASP238 and ASP239 were commonly recognized by all three ligands, frequently forming hydrogen bonds or salt bridges, and played a central role in maintaining the binding stability of the complexes. ASN167 also contributed significantly to polar recognition, especially in compounds **1** and **2**, which formed stable hydrogen bonds. In addition, PHE170 and VAL159 were involved in π‐related interactions in compounds **1** and **7**, enhancing spatial complementarity between the ligands and the binding site.

In contrast, compound **8** demonstrated a distinct binding pattern by primarily interacting with residues such as LEU84 and MET161, suggesting an alternative anchoring strategy. These differences reflect the structural diversity of the ligands and their varied recognition modes toward the target, providing valuable structural and theoretical insights for subsequent structure–activity relationship (SAR) analysis and lead compound optimization.

**Figure 6:**
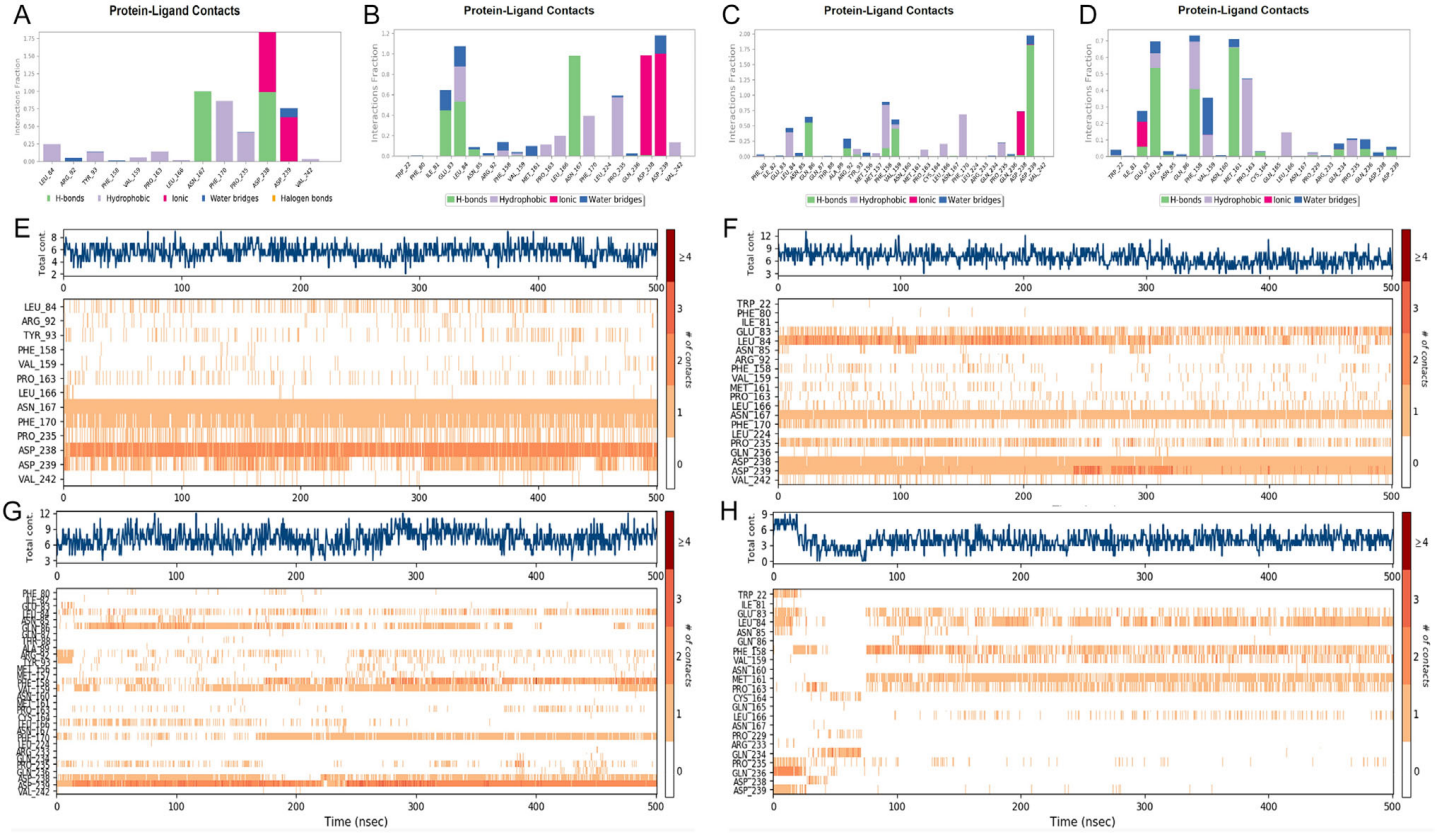
Interaction frequency profiles and contact dynamics of GPRC5D complexes with compounds **1**, **2**, **7**, and **8** during 500 ns MD simulations. A–D) Bar plots depicting the frequencies of hydrogen bonds, hydrophobic interactions, ionic contacts, and water bridges between each compound and protein residues. Panels A–D correspond to (A) compound **1**, (B) compound **2,** (C) compound **7,** and (D) compound **8**, respectively. E–H) Contact heatmaps and total contact number plots for each complex: (E) compound **1,** (F) compound **2,** (G) compound **7,** and (H) compound **8**. Blue lines indicate the total number of contacts per simulation frame, while the heatmaps display residue‐specific contact frequencies, with darker colors representing more persistent interactions.

To further validate the reliability of the static binding modes described above, dynamic interaction analyses were performed for compounds **1**, **2**, **7**, and **8** over the entire 500 ns MD simulation (**Figure** **6**). The protein−ligand interaction frequency histograms (Figure 6A–D) reveal the involvement of key residues throughout the simulation trajectory, not only confirming the core anchoring residues observed in the 2D and 3D binding mode analyses (such as ASP238, ASP239, and ASN167), but also highlighting their sustained engagement in dynamic conditions.

The contact heatmaps and total contact number plots (Figure 6E–H) provide further insights into the temporal dynamics of protein−ligand interactions. These heatmaps clearly illustrate the persistence and fluctuation of key residue contacts across the simulation timeline. For example, in the compound **1** system, residues such as ASP239 and PHE170 maintain continuous contact throughout the entire trajectory, suggesting a robust and long‐lasting anchoring effect. In contrast, compound **8** displays a more focused interaction pattern, where key residues like LEU84 and MET161 engage in highly concentrated, yet temporally segmented contacts, reflecting localized flexibility and a potentially adaptive binding mode.

To further analyze the dynamic changes and persistence of hydrogen bond formation between the ligand and GPRC5D, the VMD software was used to calculate the hydrogen bond occupancy. The analysis results are shown in Figure S10 and S11, Supporting Information. Compound **7** exhibited the highest hydrogen bond occupancy rate (83.6%), followed by compound **1** (56.3%), compound **2** (38.1%), and compound **8** (35.4%). These results not only align with the stability trends revealed by RMSF but also provide quantitative evidence for the strength and persistence of interactions between ligands and receptors.

### 3.5 ABFE Prediction for Selected Compounds

Based on ABFE calculations, the binding free energies of the four candidate compounds with GPRC5D were as follows: −9.0 kcal mol^−1^ for compound **2**, −8.9 kcal mol^−1^ for compound **7**, −8.7 kcal mol^−1^ for compound **8**, and −8.0 kcal mol^−1^ for compound **1**. Among them, compound 2 exhibited the most favorable binding affinity, suggesting a more stable thermodynamic interaction with the target. While compound **7** and compound **8** showed slightly lower predicted affinities, their values were still indicative of strong binding potential.

Although experimental validation was not performed in this study, comparative analysis of scoring, dynamic behavior, and chemical properties highlights the distinct strengths of each candidate. Compound **2** consistently ranked highest in both MM/GBSA (Δ*G* = −79.8 kcal mol^−1^) and ABFE, suggesting superior stability and binding strength. Compound **1**, while showing the lowest ABFE among the four, achieved the highest QED score (0.680), reflecting favorable drug‐likeness and potential for further development. Compound **7** shared key polar and π–π interactions with residues ASP238 and PHE170, contributing to its stable binding conformation. In contrast, compound **8** adopted a distinct binding orientation, anchored mainly by LEU84 and MET161, indicating an alternative recognition strategy within the binding pocket.

Together, these results provide diverse molecular insights into the binding behaviors of the screened hits and form a rational foundation for future SAR studies and lead compound optimization.

### 3.6 Structural Insight and Lead Compound Prioritization

Among the four prioritized compounds, compound 2 was ultimately selected as the most promising candidate based on an integrative multiparameter evaluation. This compound consistently ranked at the top across all major screening criteria: it exhibited the lowest MM/GBSA binding free energy (Δ*G* = −79.8 kcal mol^−1^), the most favorable ABFE (ABFE = −9.0 kcal mol^−1^), and a high drug‐likeness score (QED = 0.670), indicating a good balance between binding affinity and pharmacokinetic potential. Additionally, compound 2 showed stable dynamic behavior in the 500 ns MD simulations, with low RMSD fluctuations and persistent hydrogen bond interactions with key active site residues.

Detailed interaction analysis further underscored the superior binding mode of compound 2. It forms stable hydrogen bond interactions with Glu83 and Asn167, while also forming salt bridges with Asp238 and Asp239. These polar interactions collectively anchored the ligand within the hydrophilic region of the binding pocket and contributed substantially to complex stability. The combination of strong polar contacts, favorable spatial accommodation, and optimal physicochemical properties supports compound 2 as the most structurally and thermodynamically promising lead compound. These findings provide a solid foundation for its experimental validation and future optimization through SAR studies.

## 4 Conclusion

GPRC5D has recently gained significant attention in the field of cancer immunotherapy, particularly as a potential therapeutic target for hematologic malignancies such as MM.^[^ ^10^ ^,^ 39, 40, 41 ^]^ In this study, we developed an integrative virtual screening workflow to identify potential small‐molecule inhibitors targeting GPRC5D, a promising therapeutic target for MM. Through PLANET, Vina‐GPU, MM/GBSA, ADMET profiling, and rigorous MD simulations, we successfully prioritized four candidate compounds—compounds **1**, **2**, **7**, and **8**—for in‐depth analysis.

Among these, compound **2** emerged as the most promising inhibitor, exhibiting the strongest binding affinity (MM/GBSA ΔG = −79.8 kcal/mol, ABFE = −9.0 kcal/mol) and favorable drug‐like characteristics (QED = 0.670). It maintained stable hydrogen bonding interactions with key residues ASP238 and ASP239 throughout the 500 ns MD simulation, supporting a robust and thermodynamically favorable binding mode.

Compound **1**, while displaying a slightly weaker predicted binding free energy (MM/GBSA Δ*G* = −77.0 kcal mol^−1^, ABFE = −8.0 kcal mol^−1^), achieved the highest QED score (0.680), indicating excellent drug‐likeness. Its stable interactions with ASP239 and ASN167 also suggest potential for optimization and further development.

Compound **7** demonstrated unique advantages in interaction persistence, showing the highest hydrogen bond occupancy (83.6%) and strong π–π interactions with residues such as PHE170. Its ABFE value (−8.9 kcal mol^−1^) and stable MD trajectory further support its candidacy as a viable inhibitor.

Compound **8** presented a distinct binding mode, relying more on hydrophobic interactions with LEU84 and MET161, rather than the polar ASP238/239 interactions observed with other compounds. Although its hydrogen bond occupancy was lower (35.4%), it maintained high binding affinity (MM/GBSA Δ*G* = −77.8 kcal mol^−1^, ABFE = −8.7 kcal mol^−1^), offering an alternative structural scaffold for potential optimization.

Collectively, our results reveal distinct binding modes and favorable drug‐like characteristics of compounds **1**, **2**, **7**, and **8**, offering valuable molecular insights into GPRC5D–ligand interactions. These insights provide a solid framework for future SAR studies and experimental validation. However, a key limitation of this study is the absence of any known small‐molecule inhibitors or cocrystallized ligands for GPRC5D, which prevents the use of conventional benchmarking strategies such as redocking or docking score calibration with positive controls. Given that all docking scores are inherently relative, this constraint hinders the direct interpretation of absolute binding affinity. To overcome this challenge, we implemented a multilayered virtual screening strategy combining deep learning–based affinity prediction (PLANET), GPU‐accelerated molecular docking (Vina‐GPU), MM/GBSA binding energy calculations, ADMET profiling, and long‐timescale MD simulations. This integrative approach enabled internally consistent prioritization of candidates despite the lack of a reference ligand. Taken together, our workflow not only compensates for the absence of benchmark compounds but also demonstrates the feasibility of discovering novel GPRC5D‐targeted inhibitors through computational means, providing a rational starting point for further optimization and drug development.

## Conflict of Interest

The authors declare no conflict of interest.

## Author Contributions

**Xi Chen**: designed the research plan, conducted the main experiments, and contributed to manuscript writing; Xinle Yang: conducted the main experiments and contributed to manuscript writing; **Roufen Chen**: assisted in experimental design and performed dynamic simulations; **Lei Xu**: methodology, writing—original draft; **Xiaowu Dong**: supervised the research design, methodology and overall project execution; **Zhen Cai**: approved the final version of the manuscript for publication. **Xi Chen** and **Xinle Yang** contributed equally to this work.

## Supporting information

Supplementary Material

## Data Availability

The data that support the findings of this study are available from the corresponding author upon reasonable request.
