# Supplementary material for: Discovery of Potential GPRC5D Inhibitors through Virtual Screening and Molecular Dynamics Simulations
Source: ChemistryOpen. 2025 Sep 7;14(12):e202500360. doi: 10.1002/open.202500360 (PMC12680577; doi:10.1002/open.202500360)
Supplement: Supplementary file 1 — Supplementary Material [file OPEN-14-e202500360-s001.pdf]

Supplementary Information

Discovery of Potential GPRC5D Inhibitors through virtual screening and Molecular Dynamics Simulations

Xi Chen[1, 4†], Xinle Yang[3†], Roufen Chen[2], Lei Xu[5], Xiaowu Dong[2*], Zhen Cai[1,6*]

[1] Bone Marrow Transplantation Center, the First Affiliated Hospital, Zhejiang University School of Medicine, Hangzhou, Zhejiang 310003, China; chenxi2298@zjcc.org.cn

[2] College of Pharmaceutical Sciences, Zhejiang University, Hangzhou 310058, China; 12319021@zju.edu.cn (R.C.)

[3] College of Pharmaceutical Sciences, Zhejiang University of Technology, Hangzhou 310014, China; 221124070197@zjut.edu.cn (X.Y.)

[4] Lymphoma Department, Zhejiang Cancer Hospital, Hangzhou, Zhejiang 310022, China

[5] Institute of Bioinformatics and Medical Engineering, School of Electrical and Information Engineering, Jiangsu University of Technology, Changzhou, 213001, P.R. China; leixu@jsut.edu.cn (L.X.)

[6] Institute of Hematology, Zhejiang University, Hangzhou, Zhejiang 310058, China

[†]These authors contributed equally to this work.

[*]These authors are co-correspondence: Zhen Cai: caiz@zju.edu.cn; Xiaowu Dong: dongxw@zju.edu.cn.

Table of Contents

Predicted pocket locations using Schrödinger's SiteMap module. S1

Radar chart of the physical and chemical properties of compounds obtained by screening. S1-S7

Root mean square deviation (RMSD) of the GPRC5D protein backbone over a 500-nanosecond molecular dynamics (MD) simulation. S8

Root mean square fluctuation (RMSF) of the Cα atoms of GPRC5D over a 500-nanosecond molecular dynamics simulation. S9

Changes in the number of hydrogen bonds in molecular dynamics simulations of compounds 1, 2, 7, and 8. S10

The percentage of hydrogen bonds formed by four compounds in molecular dynamics simulations. S11

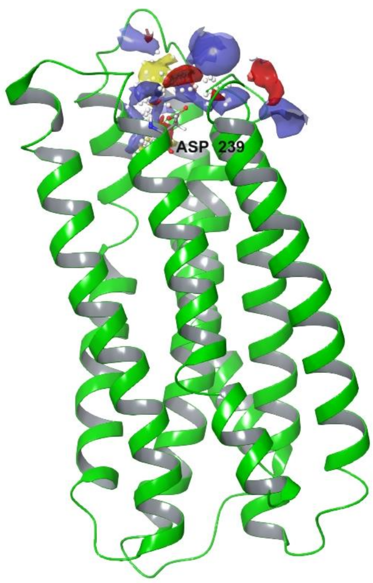

Figure S1. Predicted pocket locations using Schrödinger's SiteMap module.

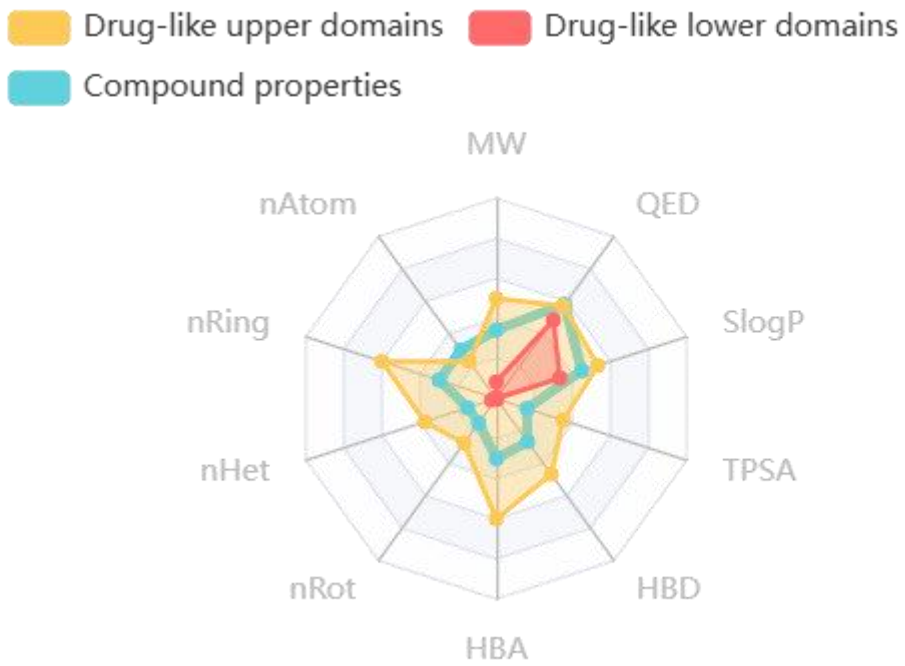

Figure S2. Radar chart of the physical and chemical properties of compound **3** obtained by screening.

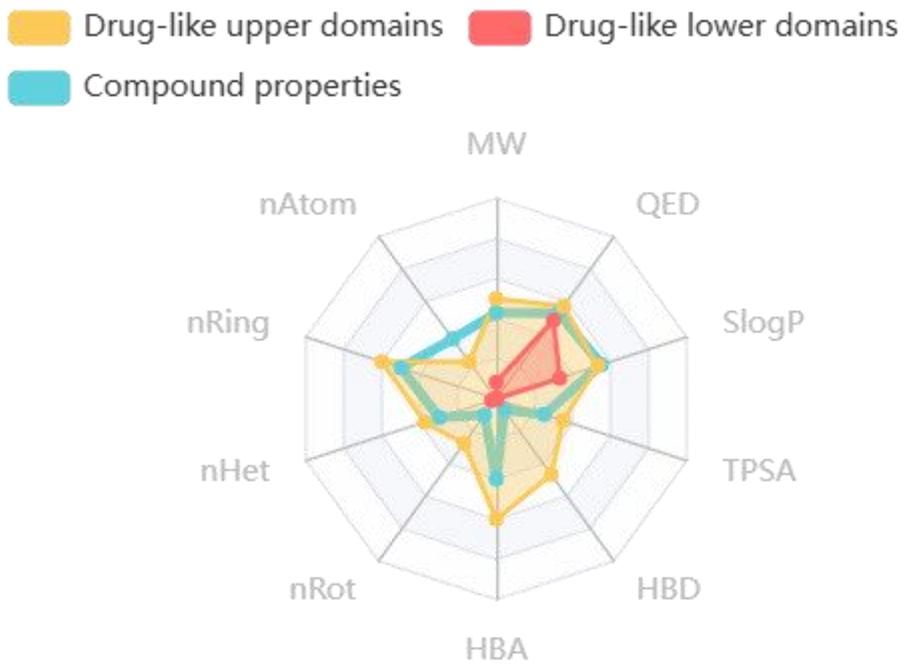

Figure S3. Radar chart of the physical and chemical properties of compound **4** obtained by screening.

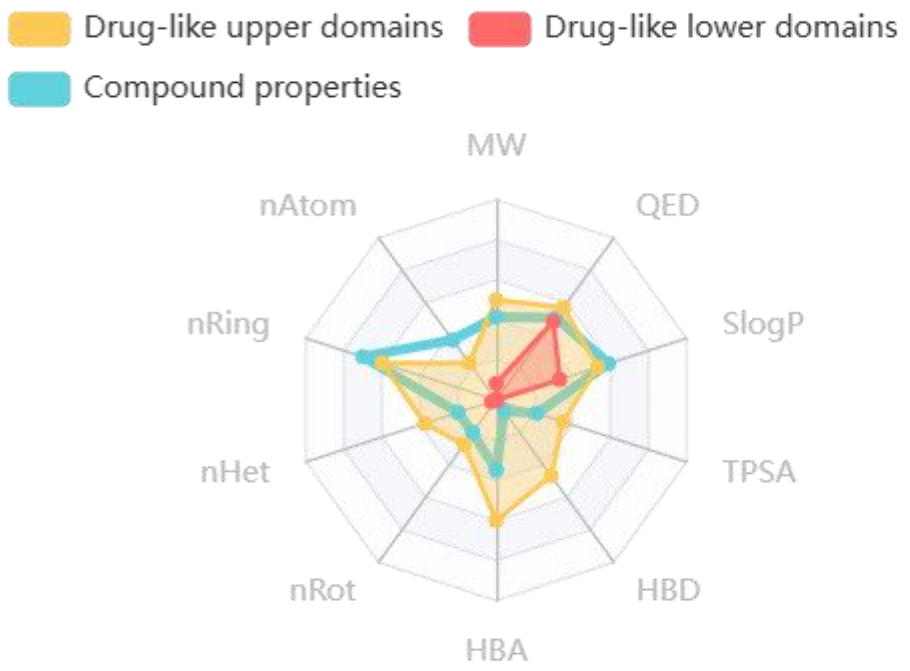

Figure S4. Radar chart of the physical and chemical properties of compound **5** obtained by screening.

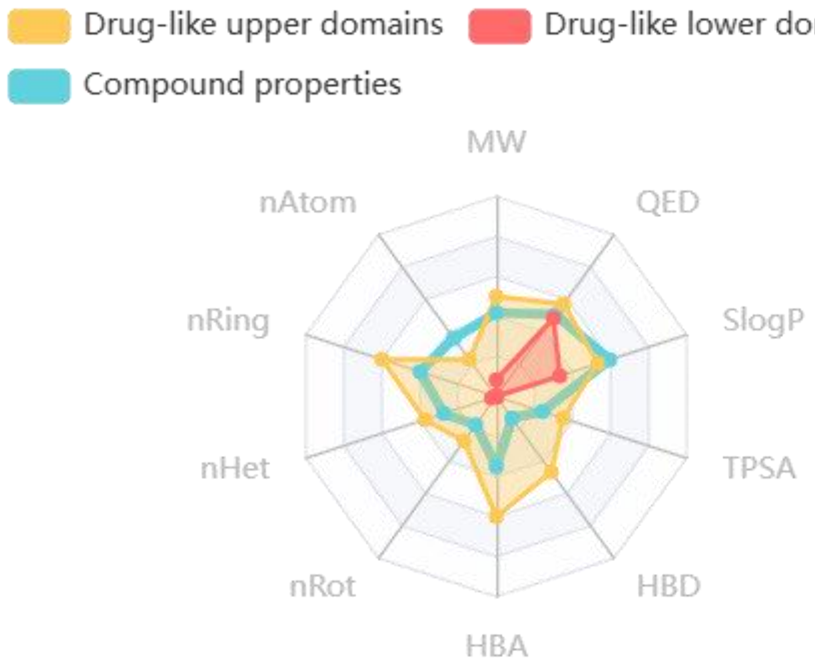

Figure S5. Radar chart of the physical and chemical properties of compound **6** obtained by screening.

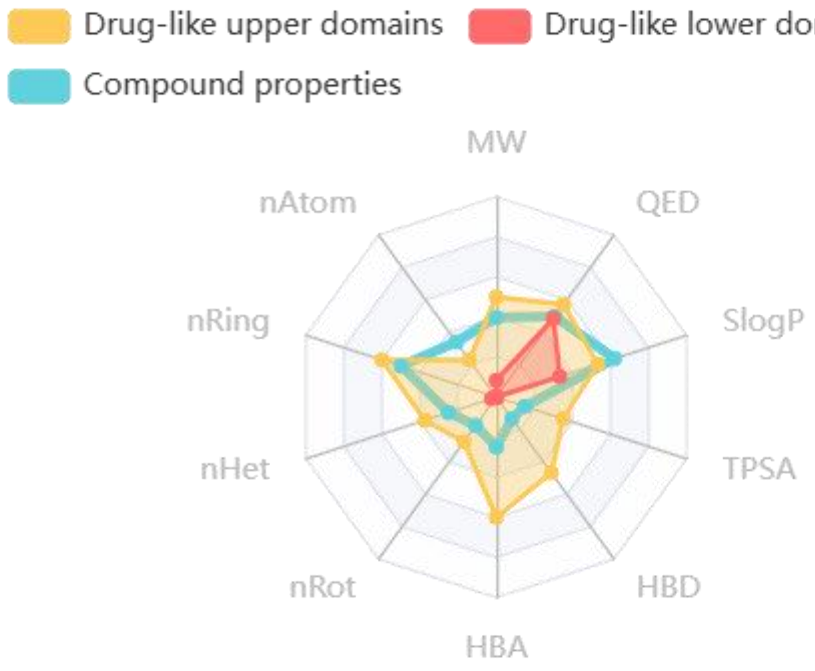

Figure S6. Radar chart of the physical and chemical properties of compound **9** obtained by screening.

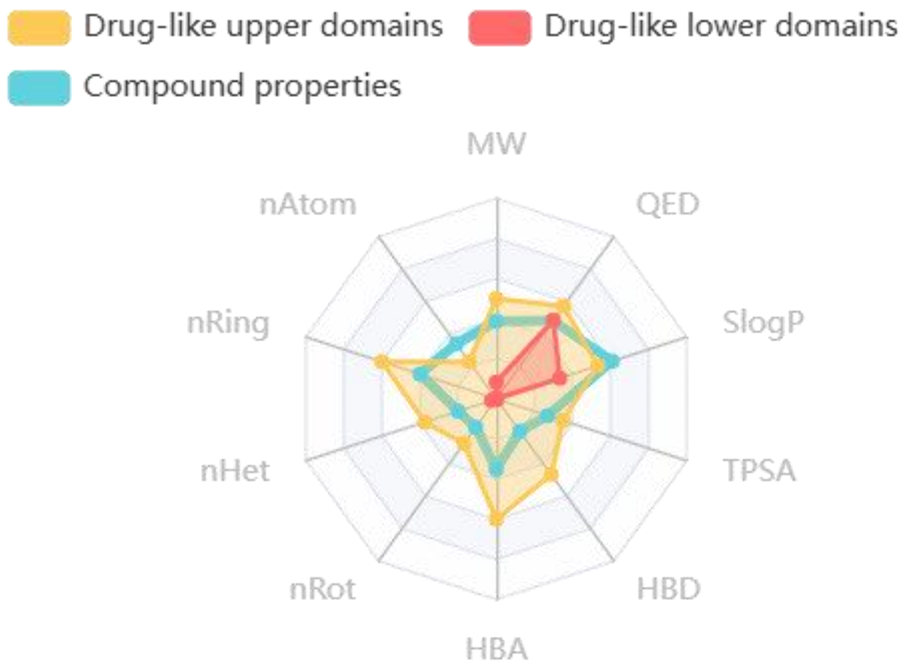

Figure S7. Radar chart of the physical and chemical properties of compound **10** obtained by screening.

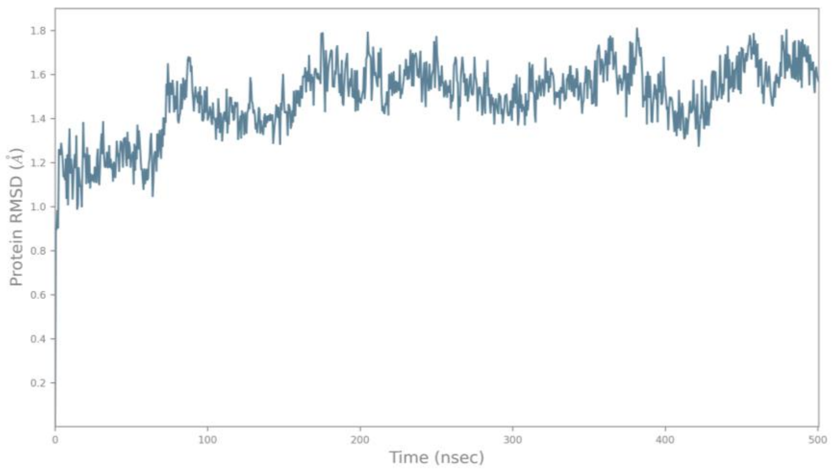

Figure S8. Root mean square deviation (RMSD) of the GPRC5D protein backbone over a 500-nanosecond molecular dynamics simulation.

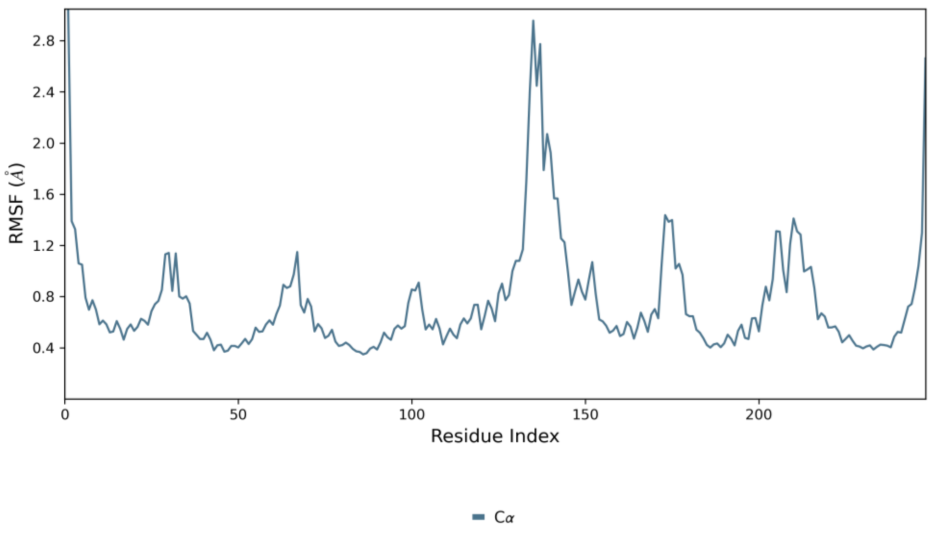

Figure S9. Root mean square fluctuation (RMSF) of the Cα atoms of GPRC5D over a 500-nanosecond molecular dynamics simulation.

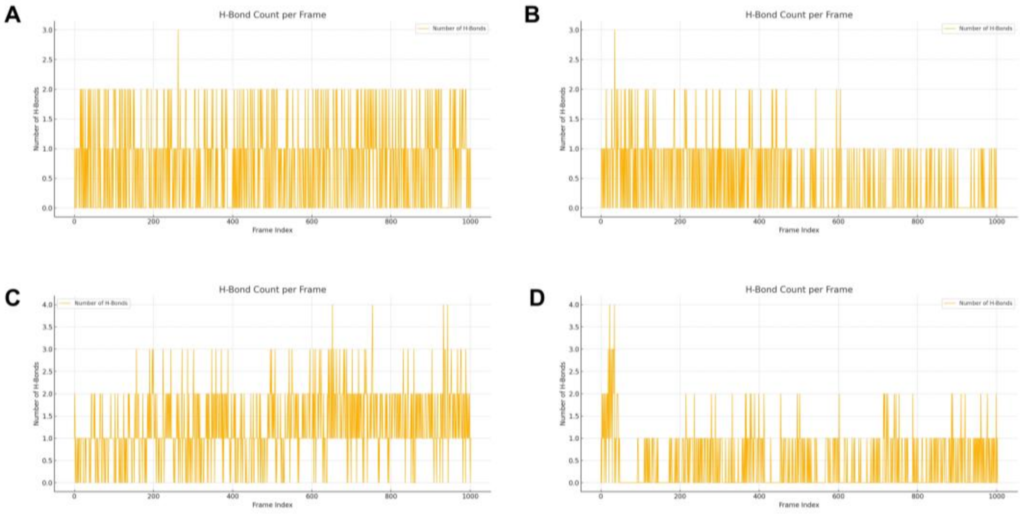

Figure S10. Changes in the number of hydrogen bonds in molecular dynamics simulations of compounds (A) **1**, (B) **2**, (C) **7**, and (D) **8**.

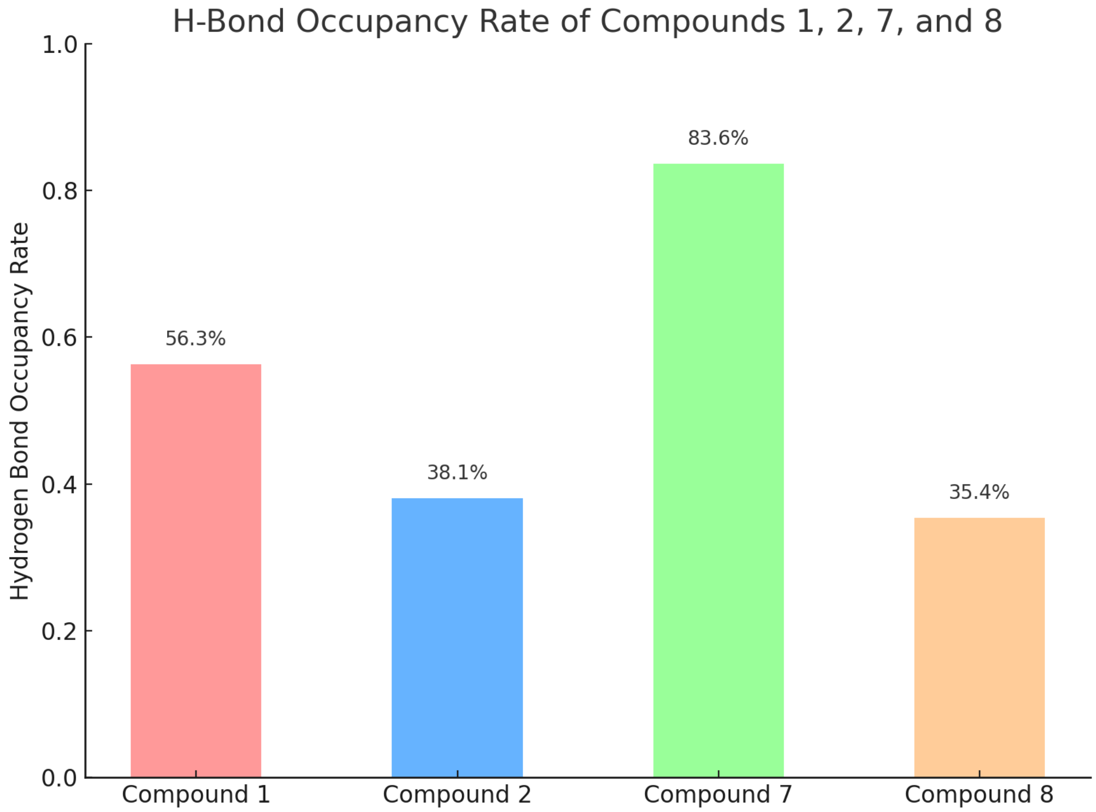

Figure S11. The percentage of hydrogen bonds formed by four compounds in molecular dynamics simulations.
